# Fractionation Mapping by Using a High-density Catheter to Map Ganglionated Plexus Sites During Sinus Rhythm

**DOI:** 10.19102/icrm.2021.120110S

**Published:** 2021-01-15

**Authors:** Jose Osorio, Douglas Mackenzie Doud, Tolga Aksu

**Affiliations:** ^1^Grandview Medical Center, Birmingham, AL, USA; ^2^Abbott, Chicago, IL, USA; ^3^University of Health Sciences, Kocaeli Derince Education and Research Hospital, Kocaeli, Turkey

**Keywords:** Cardiomyopathy, epicardial, high-density mapping, ventricular tachycardia

Ablation of the ganglionated plexi (GPs) is a relatively new technique aiming to prevent the autonomic imbalance in vasovagal syncope (VVS) from occurring. To confirm localization of the GPs, the potential usage of fragmented electrograms with visual analysis was firstly defined by our group.^1^ However, decisions made by humans based on the visual review of data may demonstrate low reproducibility.

As a new software, the fractionation mapping tool of the EnSite Precision™ cardiac mapping system was successfully used to detect GP sites during atrial fibrillation.^2^ A 18-year-old male with cardioinhibitory-type VVS was admitted with recurrent syncopal episodes despite physical counterpressure maneuvers and medications. The right and left atria were mapped with the Advisor™ HD Grid Mapping Catheter, Sensor Enabled™. Bipolar recordings were filtered at 200 to 500 Hz. A fractionation map was created using combinations of width (5 ms), refractory time (30 ms), roving sensitivity (0.1 mV), and fractionation threshold.^2^ Technical details were discussed in our previous work.^3^ White areas on the map were accepted as potential GP sites and defined as targets of ablation **(Figure 1)**. After all GP targets were identified, radiofrequency ablation of the targeted areas was initiated according to our ablation order of GPs (ie, the left superior GP, the Marshall tract GP, the left inferior GP, the right superior GP via the left atrium, and the right inferior GP via the left atrium, respectively.^4^ Radiofrequency energy application at left-sided GPs triggered a vagal response with sinus bradycardia. Meanwhile, during radiofrequency ablation at the right superior GP, the patient’s heart rate increased from 976 to 620 ms **(Video 1)**. The procedure was ended with ablation of the right superior GP via the right atrium. The final P–R interval was detected as 110 ms, which was comparable with preoperative atropine challenge results. The use of fractionation mapping software with application of the Advisor™ HD Grid mapping catheter may be used to localize GPs.

## Figures and Tables

**Figure 1:**
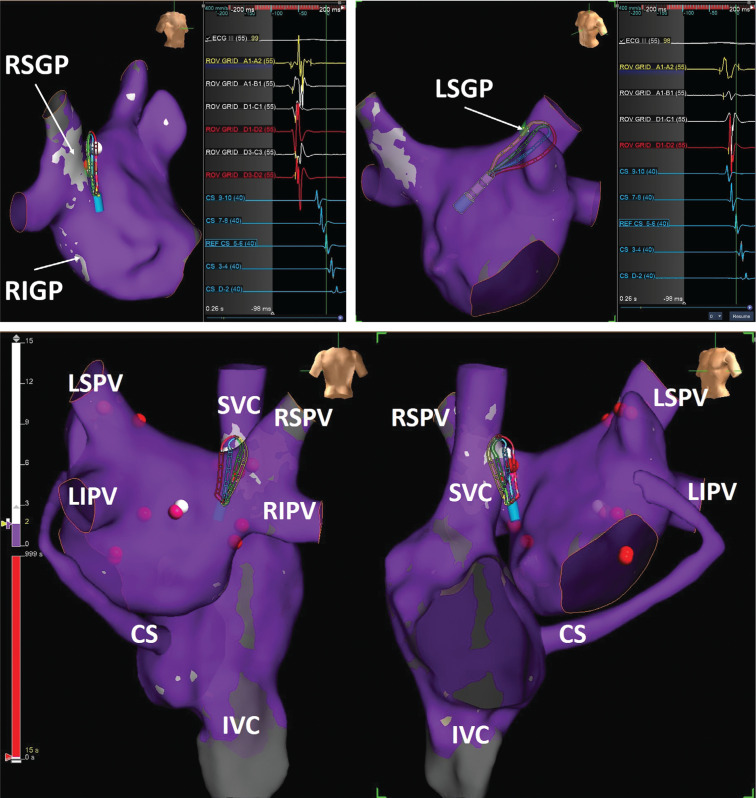
Localization of ganglionated plexuses based on fractionation mapping algorithm by using the Advisor™ HD Grid catheter.

**Video 1.** Acceleration of sinus rate is seen during radiofrequency ablation of fractionated electrograms on the right superior GP.

## References

[1] Aksu T, Guler TE, Mutluer FO, Bozyel S, Golcuk SE, Yalin K (2019). Electroanatomic-mapping guided cardioneuroablation versus combined approach for vasovagal syncope: a cross-sectional observational study.. J Interv Card Electrophysiol..

[2] Aksu T, Guler TE (2020). Electroanatomical mapping-guided ablation during atrial fibrillation: a novel usage of fractionation mapping in a case with sinus bradycardia and paroxysmal atrial fibrillation.. J Interv Card Electrophysiol..

[3] Aksu T, Guler TE, Bozyel S, Yalin K (2020). Usage of a new mapping algorithm to detect possible critical substrate for continuity of atrial fibrillation: fractionation mapping in preliminary experience. J Interv Card Electrophysiol.

[4] Armour JA, Murphy DA, Yuan BX, Macdonald S, Hopkins DA (1997). Gross and microscopic anatomy of the human intrinsic cardiac nervous system.. Anat Rec..

